# Implementation of Synoptic Reports in Enhancing Documentation Practices in Pediatric Surgical Oncology: A Systematic Review

**DOI:** 10.3390/cancers18060939

**Published:** 2026-03-13

**Authors:** Aydin Unal, Derek Harrison, Amos Hong Pheng Loh, Mohamed Albirair, Jaime Shalkow-Klincovstein, Sajid Qureshi, Simone de Campos Vieira Abib, Kokila Lakhoo, Abdelhafeez H. Abdelhafeez

**Affiliations:** 1 Department of Surgery, St. Jude Children’s Research Hospital, Memphis, TN 38105, USA; 2 Department of Pediatric Surgery, Chris Hani Baragwanath Academic Hospital, Faculty of Health Sciences, University of Witwatersrand, Johannesburg 1864, South Africa; 3 SingHealth Duke-NUS Global Health Institute, Duke-NUS Medical School, Singapore 169857, Singapore; 4 Department of Pediatric Surgery and KKH Children’s Blood and Cancer Centre, KK Women’s and Children’s Hospital, Singapore 229899, Singapore; 5 Department of Global Health, University of Washington, Seattle, WA 98195, USA; 6 Pediatric Surgery Oncology, ABC Cancer Centre, Mexico City 01120, Mexico; 7 Department of Pediatric Surgery, Tata Memorial Hospital, Mumbai 400012, India; 8 Department of Pediatric Surgery, Pediatric Oncology Institute, GRAACC, Federal University of São Paulo, São Paulo 04039-001, Brazil; 9 Nuffield Department of Surgical Sciences, Oxford University, Oxford OX3 9DU, UK; 10 Division of Pediatric Surgery, Department of Surgery, Golisano Children’s Hospital, University of Rochester Medical Center, 601 Elmwood Avenue, Box SURG, Rochester, NY 14642, USA

**Keywords:** synoptic operative reports, pediatric cancer surgery, structured documentation, pediatric surgical oncology

## Abstract

Purpose: Despite extensive evidence supporting synoptic reporting in adult surgical oncology, the pediatric surgical oncology evidence base remains sparse, institution-dependent, and implementation-limited, resulting in a critical translational gap. This systematic review evaluates the implementation and effectiveness of synoptic operative reports (SR) in improving documentation completeness in pediatric oncology surgery compared with traditional narrative reports (NR). Methods: Prospective and retrospective studies evaluating operative report completeness in pediatric oncology surgery were identified through a comprehensive search of PubMed, Scopus, and Web of Science. Of 1926 screened records, 11 articles underwent full-text review, and 4 studies met inclusion criteria. Results: The four included studies analyzed 341 operative reports (217 NRs and 124 SRs). Documentation completeness was the primary outcome. Across all evaluated intraoperative elements, synoptic reports were associated with approximately tenfold higher odds of complete documentation compared with narrative reports (pooled OR for NR vs. SR, 0.10; 95% CI, 0.07–0.14; *p* < 0.001). Conclusions: Synoptic reporting consistently improves the completeness of pediatric oncologic operative documentation compared with narrative formats; however, adoption in pediatric surgical oncology remains limited. Multicenter and implementation-focused research is needed to assess scalability, integration within electronic medical record (EMR) systems, and the impact of synoptic reporting on communication and clinical decision-making.

## Simple Summary

Operative reports are critical documents that describe what surgeons observe and perform during cancer surgery. In children with solid tumors, these details are particularly important because intraoperative findings often determine disease stage, risk classification, and treatment decisions. However, traditional narrative operative reports are written as free text and may omit important information needed by oncologists, pathologists, and multidisciplinary teams. Synoptic operative reports use structured templates that prompt surgeons to record key oncologic findings in a standardized format. In this systematic review, we evaluated existing studies comparing synoptic and narrative operative reports in pediatric surgical oncology. The available evidence shows that structured synoptic reports consistently capture more complete and clinically relevant information. These findings suggest that standardized operative documentation may improve communication, support treatment planning, and enhance the quality of surgical data used for research and international collaboration in childhood cancer care.

## 1. Introduction

Effective communication is fundamental to pediatric oncology care, where multidisciplinary coordination directly influences treatment planning and outcomes [1,2,3]. Increasing data complexity may contribute to omissions, inconsistencies, and misinterpretation within operative documentation, potentially affecting clinical decision-making [4,5,6]. Synoptic reports (SR), structured, standardized documentation tools, offers a potential solution. Originating in pathology, SRs have demonstrated improvements in clarity, completeness, and consistency of medical records and have subsequently been adopted across multiple oncologic disciplines [4,7,8,9,10,11].

Synoptic operative reporting is well established in adult surgical oncology. Numerous single-institution studies, multicenter analyses, and systematic reviews have consistently demonstrated superior documentation completeness, standardization, and data usability compared with narrative reporting, supporting widespread implementation across adult oncologic specialties and health systems [9,12,13,14,15,16].

In contrast, the pediatric surgical oncology literature remains limited, fragmented, and largely institution-specific. Pediatric malignancies differ substantially from adult cancers with respect to tumor biology, anatomic considerations, operative priorities, and protocol-driven care pathways, limiting the direct generalizability of adult evidence. Consequently, the extent to which adult synoptic reporting data have translated into pediatric surgical practice remains unclear.

Since their introduction in the 1990s, SRs have addressed key limitations of narrative reports, which frequently lack uniformity and omit critical oncologic details. The College of American Pathologists and the Royal College of Pathologists subsequently developed standardized synoptic templates incorporating essential variables such as tumor grade, resection margins, and lymph node status. These initiatives improved documentation quality, enhanced staging accuracy, and facilitated treatment planning and clinical trial enrollment [11].

In pediatric oncology, barriers to SR implementation are amplified by tumor heterogeneity and protocol-driven variability in surgical management [17,18,19,20]. Critical intraoperative findings, including tumor spillage and lymph node assessment, are frequently underreported. Existing reviews have largely focused on adult populations and demonstrate substantial heterogeneity in definitions and outcomes [4,7,10]. As a result, the pediatric evidence base supporting synoptic operative reporting has not been comprehensively evaluated.

This systematic review therefore aims not only to compare documentation completeness between synoptic and narrative operative reports in pediatric oncology surgery, but also to critically appraise the scope, independence, and maturity of the pediatric evidence base and to identify key barriers to broader implementation.

## 2. Methods

### 2.1. Search Methodology and Selection

This systematic review was conducted in accordance with PRISMA guidelines (Supplementary Table S1). The protocol was prospectively registered with PROSPERO (CRD42024595527).

### 2.2. Eligibility Criteria

Studies were eligible if they included pediatric patients undergoing resection of primary solid tumors and evaluated operative report completeness. Synoptic reports were defined as structured, standardized documentation formats, whereas narrative reports consisted of free-text documentation. Both retrospective and prospective study designs were eligible. Case reports, editorials, and studies lacking extractable quantitative completeness data were excluded.

### 2.3. Primary Outcome

The primary outcome was documentation completeness, defined as the proportion of operative reports documenting six predefined intraoperative elements: completeness of resection, intraoperative tumor spillage, locoregional involvement, vascular involvement, lymph node sampling, and specimen identification.

### 2.4. Search Strategy

A comprehensive search of PubMed, Scopus, and Web of Science was conducted using keywords related to operative reporting, synoptic documentation, pediatric surgery, and oncology. No date or language restrictions were applied. The search was completed on 15 October 2024, and updated before final analysis. Reference lists of included studies were manually screened.

### 2.5. Study Selection and Data Extraction

Two reviewers independently screened titles, abstracts, and full-text articles. Discrepancies were resolved by consensus, with third-reviewer adjudication when necessary. Extracted data included study design, sample size, tumor type, reporting format, and raw completeness data for each documentation element.

### 2.6. Statistical Analysis

For the two comparative studies, defined as studies that directly evaluated SR and NR within the same study population, raw data for each predefined intraoperative documentation element were extracted directly from the original publications. For each element, pooled 2 × 2 contingency tables were constructed comparing NR with SR, classifying reports as complete or incomplete.

Odds ratios (ORs) with 95% confidence intervals (CIs) were calculated from these pooled tables to estimate the association between report format and documentation completeness. ORs were calculated using the cross-product ratio, and 95% CIs were derived using the standard error of the log odds ratio. Statistical significance was assessed using two-sided Wald tests, with a *p* value < 0.05 considered statistically significant.

Given the limited number of comparative studies and the availability of complete raw data, pooled estimates were calculated directly from aggregated contingency tables rather than through formal study-level meta-analysis.

For non-comparative studies, defined as those evaluating narrative reports without a synoptic comparator, documentation completeness was summarized descriptively as proportions.

### 2.7. Risk of Bias

Risk of bias was assessed using the ROBINS-I tool across seven domains. Overall risk was determined by the highest-risk domain. Assessments were performed independently by two reviewers, with discrepancies resolved by consensus.

## 3. Results

### 3.1. Study Selection

**Figure 1:**
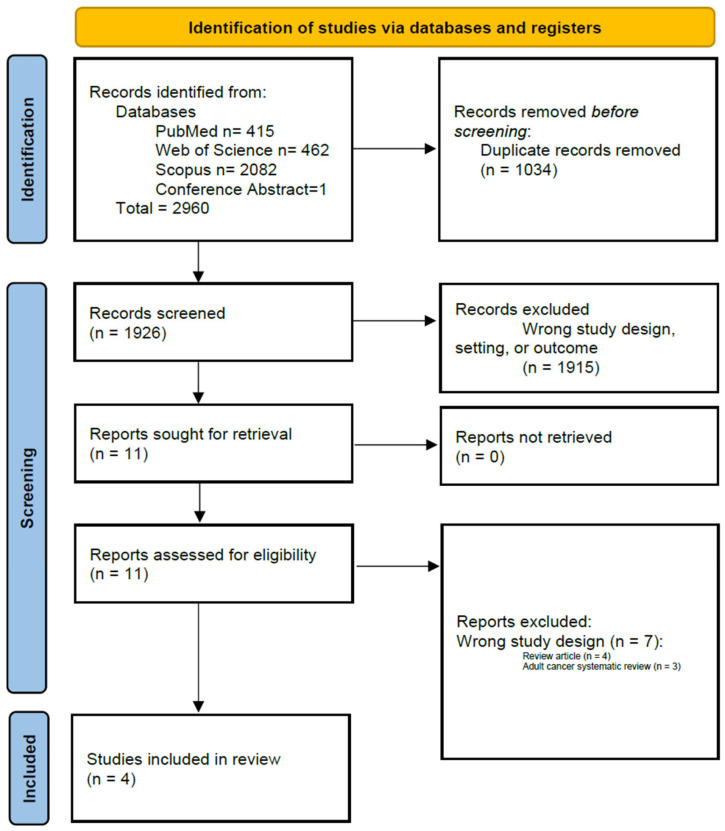
PRISMA flowchart.

Of 1928 screened records, 11 articles underwent full-text review, and 4 studies met inclusion criteria (Figure 1), highlighting the limited pediatric-specific evidence relative to the extensive adult literature. Two studies directly compared synoptic and narrative reports [21,22], whereas two evaluated narrative reporting alone [23,24], reflecting early and uneven adoption of synoptic reporting in pediatric surgical oncology.

### 3.2. Study Characteristics

Morrison et al. evaluated 35 NRs for Wilms tumor at a single institution [23]. Petrushkevich et al. analyzed 71 NRs across multiple pediatric solid tumors [24]. Abdelhafeez et al. (2024) compared 38 NRs with 32 SRs in pediatric solid tumors [21], and Abdelhafeez et al. (2026) reported a multicenter implementation study including 165 operative reports (73 NRs; 92 SRs) [22] (Table 1).

Across studies, criteria used to assess documentation completeness varied. One study applied tumor-specific criteria [24], whereas three employed hybrid frameworks incorporating universal oncologic elements alongside tumor-specific variables [21,22,23].

### 3.3. Completeness of Operative Reports

#### 3.3.1. Comparative Studies

**Figure 2:**
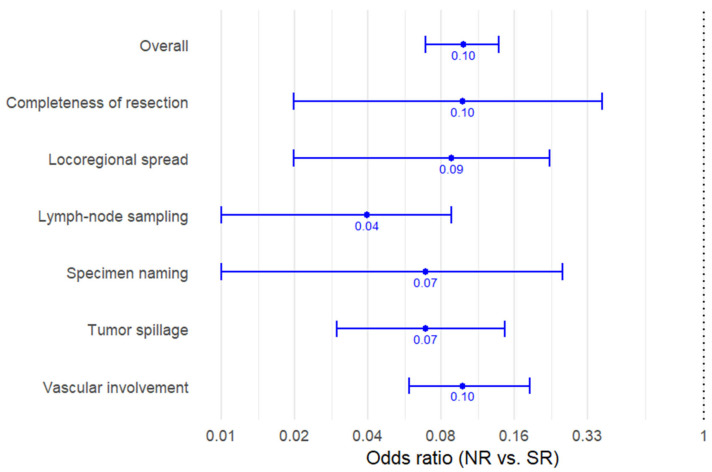
Forest plot of documentation completeness comparing narrative and synoptic operative reports in pediatric surgical oncology. Odds ratios (ORs) < 1 indicate lower odds of complete documentation with narrative reports compared with synoptic reports. Estimates were calculated directly from raw data extracted from two comparative studies. Horizontal lines represent 95% confidence intervals.

The two comparative studies included 235 operative reports (111 NRs; 124 SRs). Across all evaluated intraoperative elements, SRs demonstrated substantially higher documentation completeness than NRs (Table 2, Figure 2).

Completeness of resection was documented in 86.3% of NRs compared with 98.3% of SRs (OR, 0.10; 95% CI, 0.02–0.38; *p* = 0.003). Tumor spillage documentation increased from 48.6% in NR to 92.7% in SR (OR, 0.07; 95% CI, 0.03–0.15; *p* < 0.001). Similar improvements were observed for locoregional involvement, vascular involvement, lymph node sampling, and specimen identification.

Overall, synoptic reporting was associated with approximately tenfold higher odds of complete documentation across all intraoperative elements (pooled OR, 0.10; 95% CI, 0.07–0.14; *p* < 0.001). In absolute terms, completeness was 1.5- to 3-fold higher with SR, with the largest gains observed for tumor spillage and lymph node sampling.

One study [22] assessed usability and user perception using validated instruments, including the System Usability Scale (SUS). Synoptic reporting systems consistently achieved high SUS scores, falling within the “excellent” range and well above established acceptability thresholds.

Item-level analyses, derived from the cited study [22], demonstrated strong agreement with statements supporting frequent use, clarity, and peer recommendation. Negative-phrased items addressing complexity or excessive workload received low agreement scores, suggesting minimal perceived burden. Multidisciplinary cancer clinicians reported that synoptic reports improved clarity of operative documentation (94%), treatment planning (77%), workflow efficiency (82%), and facilitated communication (71%).

#### 3.3.2. Non-Comparative Studies

Morrison et al. reported NR completeness ranging from 26% (vascular involvement) to 100% (specimen identification), with a mean completeness of 63% across all elements [23]. Petrushkevich et al. reported overall completeness of 66% for general oncologic variables and 42% for tumor-specific variables, with marked variation by tumor type [24].

#### 3.3.3. Risk of Bias

**Figure 3:**
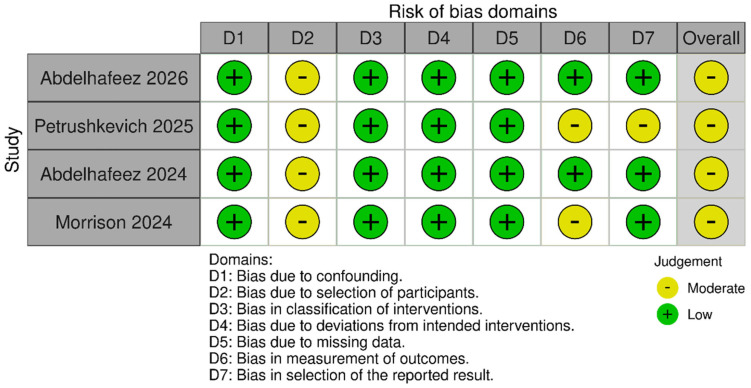
Risk of Bias Summary [21,22,23,24].

All included studies were rated as having moderate risk of bias, primarily due to observational design, tumor heterogeneity, and variable definitions of completeness (Figure 3).

## 4. Discussion

This systematic review demonstrates that synoptic operative reporting substantially improves documentation completeness in pediatric surgical oncology compared with traditional narrative reporting. Across studies, synoptic reports consistently captured critical intraoperative oncologic elements more reliably, particularly variables central to staging and protocol-driven treatment decisions such as tumor spillage and lymph node assessment. These findings are consistent with the extensive literature from adult surgical oncology demonstrating that structured operative documentation improves completeness, clarity, and consistency of surgical record [2,3,7,8,10,11,25,26,27,28,29,30,31].

However, the present review highlights an important distinction between adult and pediatric evidence. While the benefits of synoptic reporting have been extensively studied in adult malignancies, the pediatric literature remains limited in both scope and implementation. Only a small number of studies have evaluated structured operative documentation in childhood cancers, and most have focused primarily on documentation completeness rather than downstream clinical impact [21,22,23,24]. Collectively, these observations suggest that the key question in pediatric surgical oncology is no longer whether synoptic reporting improves documentation, but rather how it should be designed, implemented, and evaluated to maximize clinical relevance across heterogeneous tumors, resource settings, and health systems.

Insights from the broader adult oncology literature therefore provide important context for understanding both the potential benefits and the practical challenges of implementing synoptic reporting in pediatric surgical oncology.

### 4.1. Lessons from Adult Oncology Implementation

Although literature evaluating synoptic operative reporting in pediatric surgical oncology remains limited, extensive experience from adult oncology provides important insights into both the benefits and challenges of structured reporting systems. Evidence from adult surgical oncology and cancer pathology disciplines consistently demonstrates that synoptic reporting improves documentation completeness and enhances the clarity of clinically relevant information [2,3,4,5,6,7,8,10,11,25,26,27,28,29,30,31,32,33,34,35].

In cancer pathology, several studies have shown that synoptic reporting significantly improves the completeness of diagnostic reports [4,5,6,27,32,33,34,35]. A systematic review by Sluijter and colleagues evaluating structured pathology reporting for solid tumors found that synoptic templates increased the reporting of essential tumor characteristics compared with traditional narrative reports [4]. Standardized checklists ensured that critical data elements were consistently documented, thereby improving the reliability of information used for staging and treatment planning.

Similar benefits have been observed in adult surgical oncology [2,3,7,8,10,11,25,26,27,28,29,30,31]. Studies evaluating the adoption of synoptic operative reports across multiple cancer procedures have reported improvements in documentation completeness of approximately 30% compared with narrative operative reports [7]. These improvements are particularly evident for key oncologic variables that are often inconsistently documented in free-text reports, including completeness of resection and margin assessment. By prompting surgeons to address predefined data elements, synoptic templates promote systematic documentation of essential surgical details. In addition, structured operative reports have been recognized as useful educational tools for surgical trainees by highlighting critical oncologic principles that should be assessed during procedures [8,10,36,37,38,39].

Beyond improving completeness, structured reporting may also enhance the interpretability of clinical reports for multidisciplinary teams, potentially improved risk stratification, and oncologic outcome [3,25].

Despite these advantages, the implementation of synoptic reporting systems presents several challenges. Early adoption may raise concerns regarding workflow efficiency, as some clinicians perceive structured templates to be more time-consuming than narrative dictation [5,9,12,13,15,16]. These concerns are often related to unfamiliarity with reporting interfaces or the perception that rigid templates limit narrative flexibility. Successful implementation therefore requires a careful balance between comprehensiveness and usability, ensuring that templates capture oncologically meaningful intraoperative information without imposing excessive documentation burden. Early clinician engagement, training, and advocacy during the pre-implementation phase are therefore critical to facilitate adoption [5,9,12,13,15,16].

Technical integration within electronic medical record systems represents another important barrier. Implementing synoptic templates frequently requires dedicated information technology support, and differences between electronic health record platforms may limit the portability of standardized templates across institutions. Cultural and behavioral factors may also influence adoption. Surgeons accustomed to traditional narrative dictation may initially resist structured reporting systems, particularly if they perceive them as restrictive or disruptive to established workflows. Institutional leadership support, combined with targeted training and iterative refinement of reporting templates, can help address these concerns and promote successful implementation [5,9,12,13,15,16].

Collectively, these experiences from adult oncology provide valuable lessons for pediatric surgical oncology. They highlight the importance of balancing standardization with usability, ensuring effective integration within electronic health record systems, and actively engaging clinicians in the development and refinement of reporting tools.

### 4.2. Usability as a Determinant of Sustainability

An important contribution of the pediatric synoptic reporting literature is the evaluation of usability and end-user experience. Implementation studies included in this review indicate that surgeons and oncology teams generally perceive synoptic reports as improving the clarity, efficiency, and interpretability of operative findings. High scores on the System Usability Scale (SUS), falling within the “excellent” range and exceeding commonly accepted usability thresholds, suggest that structured operative reporting can be implemented without imposing excessive cognitive or time burden on surgeons [22].

Item-level responses from usability assessments further suggest that synoptic reporting is not merely tolerated but actively preferred by users. Surgeons expressed strong willingness to use synoptic reports routinely, continue using them in future practice, and recommend them to colleagues. Importantly, items assessing perceived system complexity, workload, or technical difficulty received consistently low scores, countering a common concern that structured documentation increases administrative burden. This favorable perception likely reflects thoughtful template design in which synoptic reports focus on critical intraoperative oncologic findings while avoiding duplication of preoperative data or nonessential information that does not directly inform treatment decisions.

Despite these positive findings, improvements in multidisciplinary communication appear more modest. While clinicians reported greater clarity in operative documentation, the perceived impact on multidisciplinary team (MDT) communication was less pronounced [22]. This observation suggests that structured documentation alone may not be sufficient to optimize interdisciplinary collaboration. To fully realize its potential, synoptic reporting may need to be integrated into broader clinical information systems, including pathology synoptic reports, oncology treatment planning platforms, and shared MDT dashboards. In this context, synoptic operative reports should be viewed not simply as structured documents but as interoperable data elements within a broader oncologic information ecosystem.

### 4.3. Universal Versus Tumor-Specific Reporting: An Implementation Tension

A key insight emerging from this review is the tension between universality and specificity in synoptic operative reporting design. Most pediatric studies to date have relied on templates structured around universal oncologic principles rather than tumor-specific intraoperative variables [21,22,23]. This approach offers several practical advantages, including broad applicability across tumor types, simplified implementation, and easier integration into existing clinical documentation systems.

However, pediatric oncology surgery is characterized by substantial heterogeneity. The intraoperative findings that determine risk stratification and guide therapy differ markedly across tumor types. For example, tumor spill and margin status are critical determinants of staging in Wilms tumors and ovarian germ cell tumors, whereas the extent of tumor resection and residual disease burden are more relevant in neuroblastoma, where complete resection is often neither feasible nor required. Similarly, biopsy tract excision is an important consideration in sarcoma surgery but irrelevant in many other malignancies [40].

A purely generic synoptic template may therefore risk overlooking disease-specific variables that are essential for treatment planning. Reflecting this concern, cooperative research groups have begun developing tumor-specific synoptic operative reports. For example, the International Neuroblastoma Surgical Report Form (INSRF) was developed through a collaboration between major pediatric oncology cooperative groups to standardize reporting of neuroblastoma surgery and capture disease-specific operative variables relevant to staging and treatment planning [19]. Similarly, the Children’s Oncology Group has initiated the development of disease-specific templates, including a renal tumor synoptic operative report incorporated into the AREN2231 clinical trial [41,42]. These initiatives represent an important evolution in the role of operative documentation, positioning the operative report as an active component of protocol compliance rather than a purely retrospective clinical record.

While tumor-specific frameworks provide comprehensive capture of intraoperative findings relevant to individual diseases, they may also include elements beyond strictly oncologic intraoperative variables, such as perioperative events or preoperative imaging findings. An alternative strategy is the use of hybrid synoptic reporting templates, which focus specifically on intraoperative findings of oncologic significance that influence staging, risk stratification, and therapeutic decision-making [40]. In this model, the synoptic report captures tumor-specific oncologic variables while allowing other perioperative details to be documented through existing clinical documentation systems.

Thus, the synoptic report functions as a structured summary of critical intraoperative oncologic findings rather than a complete replacement for the traditional operative note (Supplementary Figure S1). This approach reflects an important conceptual distinction: synoptic operative reporting is intended to augment conventional documentation systems by ensuring consistent capture of key oncologic variables that directly influence multidisciplinary treatment decisions. By integrating structured oncologic reporting with existing operative documentation workflows, hybrid templates may offer a practical balance between standardization, usability, and clinical relevance.

### 4.4. Global and Resource Considerations

The design of synoptic reporting systems also has important implications for global implementation. Tumor-specific templates may maximize clinical relevance but can introduce practical challenges in real-world settings. Surgeons who manage diverse tumor types may find navigating multiple templates cumbersome, potentially reducing compliance. From an informatics perspective, maintaining and updating numerous disease-specific templates increases administrative complexity and may strain electronic medical record infrastructure.

These challenges are particularly relevant in low- and middle-income countries (LMICs), where EMR systems may be limited or absent and documentation may remain paper-based. Implementing multiple tumor-specific paper forms in such environments may be impractical and could undermine standardization efforts. A simplified synoptic template grounded in universal oncologic principles may therefore provide a more feasible entry point for structured reporting in these settings.

In many LMIC contexts, the operative report also assumes heightened clinical importance. Pathology services may be constrained by workforce shortages, delayed processing times, limited access to immunohistochemistry or molecular diagnostics, and variability in specimen handling. Under these conditions, intraoperative findings frequently play a central role in staging, risk stratification, and determination of therapy intensity. Variables such as tumor rupture, nodal assessment, gross residual disease, and vascular involvement may guide treatment decisions when detailed pathologic data are unavailable or delayed. In this environment, a structured operative report becomes not only a documentation tool but also a critical component of therapeutic decision-making [43].

Standardized synoptic reporting may therefore support equity in pediatric cancer care by improving communication across geographically distributed care teams, including regional referral centers and international collaborative programs. The studies included in this review demonstrate that synoptic reporting can be implemented across diverse healthcare environments, with high acceptance among surgeons and oncology teams [22].

### 4.5. Toward a Hybrid Model

Taken together, these findings suggest that the future of synoptic operative reporting in pediatric surgical oncology will likely involve a hybrid model. Such an approach would combine a universal core set of essential oncologic elements applicable to all pediatric solid tumors with tumor-specific modules tailored to individual malignancies.

This modular architecture offers several advantages. Universal core elements enable standardized documentation across tumor types, facilitating quality monitoring, multicenter research, and cross-institutional comparisons. Tumor-specific extensions ensure that critical disease-defining variables are captured without overwhelming clinicians with excessively complex templates.

From an implementation perspective, a hybrid structure may also reduce cognitive burden by providing surgeons with a familiar documentation framework while allowing disease-specific sections to be activated when relevant. Such an approach balances standardization with flexibility and may represent the most scalable strategy for global adoption.

Importantly, synoptic reporting systems are generally designed to complement rather than replace narrative operative documentation. Structured data fields capture key oncologic findings in a standardized format, while optional free-text sections preserve the ability to document complex operative nuances and contextual details that may not be fully represented within predefined fields. This combined structure allows synoptic reports to support both standardized data capture and the clinical richness traditionally provided by narrative operative notes.

### 4.6. Synoptic Operative Reporting as Structured Data Infrastructure

Beyond improving documentation completeness, synoptic operative reporting has important implications for the development of structured clinical data infrastructure in pediatric oncology. Operative reports traditionally function as narrative clinical records designed primarily for communication between clinicians. However, in modern oncology systems, surgical documentation increasingly serves an additional role as a source of structured data supporting quality monitoring, clinical research, and protocol adherence [8,10,11,27,44,45].

In adult oncology, structured operative and pathology reporting has been instrumental in enabling national quality improvement initiatives and registry-based research [4]. By capturing predefined clinical variables in standardized formats, synoptic reporting facilitates reliable extraction of key oncologic parameters across institutions. This capability is particularly important in pediatric oncology, where rare diseases require large-scale collaboration to generate meaningful clinical evidence.

Childhood cancers are characterized by low incidence and substantial biological heterogeneity. As a result, treatment advances often depend on multicenter cooperative group trials and international registries. In this environment, the quality and completeness of clinical documentation directly influence the reliability of shared data. Narrative operative reports, which vary widely in structure and terminology, are poorly suited for systematic data abstraction. Important intraoperative variables may be omitted, described inconsistently, or embedded in free text that is difficult to extract for research purposes [10].

Synoptic reporting addresses this limitation by transforming operative documentation into structured datasets. When key intraoperative elements such as residual disease and nodal sampling are captured through standardized fields, they become immediately accessible for quality monitoring and research analysis. This approach reduces the need for retrospective chart review and improves the accuracy of data submitted to registries and clinical trial databases [8,10,11,27,44,45].

The growing integration of synoptic operative reports within cooperative group protocols illustrates this emerging role. For example, disease-specific synoptic templates are increasingly incorporated into prospective clinical trials to ensure consistent documentation of protocol-relevant surgical variables [41,42]. In this context, the operative report becomes not only a clinical record but also a structured instrument supporting trial integrity. Standardized documentation helps ensure that staging variables and surgical endpoints are captured uniformly across participating institutions, reducing variability that may otherwise confound study results.

Structured operative reporting also creates opportunities for automated data integration within electronic health record systems. When operative findings are recorded using discrete data elements, these variables can be linked with pathology reports, imaging data, and treatment plans to form comprehensive oncology datasets. Such integration could support real-time clinical decision support, facilitate automated staging calculations, and enable dashboards for multidisciplinary tumor boards [8,10,11,27,44,45].

The potential value of structured surgical data extends further into emerging areas of digital health and artificial intelligence [45]. Large, standardized datasets derived from synoptic operative reports could support machine learning models designed to predict surgical outcomes, identify risk factors for complications, or optimize treatment pathways [8,45]. Although such applications remain largely conceptual within pediatric oncology, they underscore the importance of establishing standardized documentation frameworks that enable high-quality data generation.

Importantly, the benefits of structured operative data are not limited to highly resourced healthcare systems. In settings where formal oncology registries or advanced informatics infrastructure are limited, standardized synoptic reports may provide a practical mechanism for improving data quality and enabling participation in international collaborative research. By reducing variability in operative documentation, synoptic reporting can facilitate more reliable communication between institutions and strengthen global pediatric cancer networks [45].

Viewed through this lens, synoptic operative reporting represents more than a documentation tool. It constitutes a foundational component of the data ecosystem required to support modern pediatric oncology care. As treatment protocols become increasingly complex and data-driven, the ability to capture accurate intraoperative information in structured formats will become progressively more important. Future implementation efforts should therefore consider synoptic reporting not only as a means of improving operative notes but also as a strategy for building the clinical data infrastructure necessary to advance pediatric cancer research and quality improvement.

### 4.7. From Documentation Completeness to Clinical Relevance

Operative documentation plays a particularly critical role in pediatric oncology, where intraoperative findings directly influence staging, risk stratification, and eligibility for cooperative group treatment protocols. In contrast to many adult cancers where staging is frequently driven by preoperative imaging or pathologic parameters, pediatric treatment algorithms often rely heavily on intraoperative variables that cannot be reconstructed retrospectively [46,47]. As a result, omissions or ambiguities in operative reports may lead to misclassification of disease stage, uncertainty during multidisciplinary tumor board discussions, or deviations from protocol-based therapy.

The studies included in this review consistently demonstrate that narrative reports incompletely capture many of these essential intraoperative elements [21,22,23,24]. Importantly, in the comparative studies, predefined oncologic documentation elements were operationalized a priori and applied uniformly to both narrative and synoptic reporting formats. This methodological approach ensured that differences in documentation completeness reflected the reporting structure rather than subjective interpretation of operative findings. The results suggest that narrative documentation most frequently fails to capture tumor-specific staging variables rather than general descriptive information, highlighting an inherent limitation of free-text reporting in capturing protocol-relevant surgical data.

Nevertheless, improved documentation completeness should not be interpreted as an endpoint in itself. Documentation quality represents a surrogate outcome; its ultimate value depends on whether it improves clinical decision-making, multidisciplinary communication, and patient outcomes [3,25]. This distinction is particularly important in pediatric surgical oncology, where operative documentation functions not only as a clinical record but also as a source of structured data supporting protocol adherence, quality assurance, and international collaborative research.

### 4.8. Evidence Gaps and Future Research Directions

Despite clear evidence that synoptic reporting improves documentation completeness, several important knowledge gaps remain. Most notably, the relationship between improved documentation and clinical outcomes has not yet been established. Future research should examine whether structured operative reporting improves staging accuracy, reduces protocol deviations, enhances adherence to surgical guidelines, or ultimately influences recurrence and survival outcomes.

Understanding the root causes of documentation gaps in narrative reporting is also critical. Factors such as time pressure, workflow inefficiencies, lack of standardized training, and limitations in electronic documentation systems may contribute to incomplete reporting. Qualitative research exploring surgeon perspectives could provide valuable insights into barriers to adoption and inform implementation strategies that align with real-world clinical workflows.

Finally, implementation research in resource-limited settings remains a priority. Evaluating simplified synoptic templates, paper-based systems, or combined digital-paper approaches may help ensure that the benefits of structured documentation extend beyond highly resourced institutions. Cost-effectiveness analyses and sustainability studies will also be essential to support long-term adoption.

### 4.9. Limitations

This review has several limitations. The number of comparative studies was small, reflecting the early stage of synoptic reporting implementation in pediatric surgical oncology. Comparative data were derived from a limited number of institutions, underscoring the need for independent multicenter validation. Additionally, most studies were observational in design, and improvements in documentation completeness do not necessarily translate into improved clinical decision-making or patient outcomes.

## 5. Conclusions

This systematic review confirms that synoptic operative reporting substantially improves documentation completeness in pediatric surgical oncology, particularly for intraoperative elements critical to staging and treatment planning. However, the pediatric evidence base remains narrow and focused primarily on implementation rather than clinical outcomes.

The central challenge moving forward is not demonstrating that synoptic reporting improves documentation, but designing systems that balance standardization, tumor-specific relevance, usability, and scalability across diverse healthcare environments. A hybrid synoptic reporting model incorporating a universal core with tumor-specific extensions represents a pragmatic and forward-looking strategy. Continued international collaboration, integration with cooperative group trials, and rigorous evaluation of downstream clinical impact will be essential to fully realize the potential of synoptic reporting as a tool for improving pediatric cancer care.

## Figures and Tables

**Table 1:** Characteristics of the included study.

Author (Year)	Population	Study Type	NR	SR	Primary Outcome
Morrison 2024 [23]	Wilms tumors	Single center retrospective NR	35	0	Inclusion of 6 key intraoperative findings
Petrushkevich 2025 [24]	Four pediatric solid tumors	Single center retrospective NR	71	0	Completeness of NR is measured by tumor-specific audit tools.
Abdelhafeez 2024 [21]	Pediatric solid tumors	Single center comparative	32	38	Inclusion of 6 key intraoperative findings
Abdelhafeez 2026 [22]	Pediatric solid tumors	Multicenter comparative	73	92	Inclusion of 6 key intraoperative findings

**Table 2:** Comparison of Documentation Completeness Between Narrative and Synoptic Operative Reports.

Documentation Element	NR Completeness % (Pooled *n*/N)	SR Completeness % (Pooled *n*/N)	Pooled OR (NR vs. SR) [95% CI]
Completeness of resection	86.5 (96/111)	98.4 (122/124)	0.10 [0.02–0.38]; *p* = 0.003
Tumor spillage	48.6 (54/111)	92.7 (115/124)	0.07 [0.03–0.15]; *p* < 0.001
Locoregional spread	72.1 (80/111)	96.8 (120/124)	0.09 [0.02–0.23]; *p* < 0.001
Vascular involvement	32.4 (36/111)	82.3 (102/124)	0.10 [0.06–0.19]; *p* < 0.001
Lymph-node sampling	51.4 (57/111)	96.8 (120/124)	0.04 [0.01–0.09]; *p* < 0.001
Specimen naming	82 (91/111)	98.4 (122/124)	0.07 [0.01–0.26]; *p* < 0.001
Overall (combined)	_	_	0.10 [0.07–0.14]; *p* < 0.001

## Data Availability

The data will be available on reasonable request from the corresponding author.

## Supplementary Materials

The following supporting information can be downloaded at https://www.mdpi.com/article/10.3390/cancers18060939/s1. Figure S1: Web-Based Hybrid Synoptic Operative Report. (A) Examples of universal core oncologic elements. (B) Examples of tumor-specific key elements. Table S1: PRISMA 2020 checklist.

## Abbreviations

The following abbreviations are used in this manuscript:

NRs	Narrative Reports
SRs	Synoptic Reports
