# Supplementary material for: Implementation of Synoptic Reports in Enhancing Documentation Practices in Pediatric Surgical Oncology: A Systematic Review
Source: Cancers (Basel). 2026 Mar 13;18(6):939. doi: 10.3390/cancers18060939 (PMC13024793; doi:10.3390/cancers18060939)
Supplement: Supplementary file 1 [file cancers-18-00939-s001.zip › Figure S1.pdf]

A- Examples of Universal core elements

Completeness of Resection

- Wide Local Resection, potentially R0
- Marginal Resection, potentially R1
- Debulking, R2
- Biopsy Only

Was there interaoperative tumor spillage

- Yes
- No
- N/A (e.g. Neuroblastoma)

Completeness of Resection

- Wide Local Resection, potentially R0
- Marginal Resection, potentially R1
- Debulking, R2
- Biopsy Only

Was there interaoperative tumor spillage

- Yes
- No
- N/A (e.g. Neuroblastoma)

Was there vascular encasement encountered?

- Yes, vascular encasement
- No, vascular encasement
- Tumor only abuts vessels

Was there a tumor thrombus?

- No tumor thrombus
- Tumor thrombus not resected
- Piece meal resection of tumor thrombus
- Completely resected en bloc with primary tumor
- Not applicable

Was there evidence of locally advanced disease?

- Yes
- No

For tumors with suspicious free peritoneal fluid, was ascitic fluid sampled?

- Yes
- No
- N/A

Were regional lymph nodes sampled?

- Yes
- No
- Not Indicated
- N/A

B- Examples of Tumor-specific key elements

Tumor Type

Neuroblastoma

What was the estimated percentage of tumor resection?

Sarcoma

Was the biopsy tract resected en bloc?

- Yes
- No
- N/A

Ovarian Germ Cell Tumor

Which of the following procedures were performed regarding the contralateral ovary and/or peritoneal fluid? (Select all that apply)

- [ ] Contralateral ovary was evaluated for malignancy
- [ ] Peritoneal fluid was sampled
- [ ] Neither of these procedures was performed
- [ ] N/A - primary GCT is not ovarian
